# LncRNA HOXA‐AS2 positively regulates osteogenesis of mesenchymal stem cells through inactivating NF‐κB signalling

**DOI:** 10.1111/jcmm.14034

**Published:** 2018-12-08

**Authors:** Xinxing Zhu, Jinjin Yu, Jiang Du, Genshen Zhong, Liang Qiao, Juntang Lin

**Affiliations:** ^1^ Henan Joint International Research Laboratory of Stem Cell Medicine College of Biomedical Engineering Xinxiang Medical University Xinxiang China; ^2^ Stem Cell and Biotherapy Engineering Research Center of Henan College of Life Science and Technology Xinxiang Medical University Xinxiang China; ^3^ School of Psychology Xinxiang Medical University Xinxiang China; ^4^ Henan Collaborative Innovation Center of Molecular Diagnosis and Laboratory Medicine School of Laboratory Medicine Xinxiang Medical University Xinxiang China

**Keywords:** HDAC2, HOXA‐AS2, lncRNA, NF‐κB signalling, osteogenesis

## Abstract

As is previously reported, mesenchymal stem cells have potential ability to differentiate into osteocytes. However, the underlying mechanism during this biological process is poorly understood. In the present study, we identify a novel long non‐coding RNA named HOXA‐AS2 as a critical regulator during the formation of osteogenesis. Attenuation of HOXA‐AS2 can reduce the calcium deposition and repress the alkaline phosphatase activity. Moreover, the expressions of osteogenic marker genes are markedly downregulated after HOXA‐AS2 depletion. Mechanistically, we found HOXA‐AS2 can regulate the transcriptional activity of NF‐κB, a critical inhibitor of osteogenesis. More importantly, HOXA‐AS2 knockdown could result in the transcriptional repression of the osteogenic master transcription factor SP7 by a NF‐κB/HDAC2‐coordinated H3K27 deacetylation mechanism. Based on these studies, we conclude that HOXA‐AS2 may serve as a promising therapeutic target for bone tissue repair and regeneration in the near future.

1

**Highlights**

- HOXA‐AS2 functions as a positive regulator for osteogenesis of mesenchymal stem cells.
- HOXA‐AS2 can promote osteogenesis by repressing the activity of NF‐κB signaling.
- Attenuation of HOXA‐AS2 epigenetically regulates the expression of SP7, a critical master transcription factor of osteogenic differentiation, through a NF‐κB/HDAC2‐coordinated H3K27 deacetylation mechanism.

## 2 INTRODUCTION

Mesenchymal stem cells (MSCs), a group of cells with excellent proliferation and multiple differentiation capacities, can be isolated from adipose tissue, bone marrow and many other tissues. Emerging evidence reported MSCs are able to differentiate into diverse types of cells, such as chondrocytes, osteoblasts and adipocytes, under specific conditions.^1^, ^2^, ^3^ Moreover, MSCs usually have low immunogenicity.^3^ These properties make it become an ideal option for regenerative medicine, tissue engineering and clinical therapy. The differentiation of MSCs can be regulated by specific growth factors, signalling molecules and epigenetic modifiers. More importantly, a number of phenotypic transcription factors were identified to regulate lineage commitment of MSCs, such as PPARγ and C/EBPα are involved in adipogenic differentiation,^4^, ^5^, ^6^ RUNX2 and SP7 contribute to the osteogenesis,^7^, ^8^, ^9^ Sox9 is a chondrogenic regulator.^10^, ^11^, ^12^

Inflammatory response is widely implicated in multiple cellular processes, such as proliferation, apoptosis and differentiation.^13^, ^14^, ^15^, ^16^, ^17^ It is reported inflammation is significantly associated with osteogenic differentiation of MSCs. Furthermore, as is widely accepted that activation of inflammatory response has an inhibitory effect on osteogenesis.^18^, ^19^, ^20^ A recent study revealed TNFα‐induced NF‐κB activation upregulates microRNA‐150‐3p and inhibits osteogenesis of MSCs through downregulating β‐catenin.^21^ Another study showed decreased osteogenesis in mesenchymal stem cells derived from the aged mouse is associated with enhanced NF‐κB activity.^19^ These studies demonstrated the significant correlation of NF‐κB‐mediated inflammation with osteogenesis.

Long non‐coding RNAs, a subset of non‐coding transcripts with more than 200 nucleotides in length, have been shown to play important roles in numerous biological processes, for example inflammation, immunity and various cancers.^22^, ^23^, ^24^, ^25^ With the development of high throughput RNA sequencing approach, a variety of novel lncRNAs were identified. However, due to the tissue specific and low expression properties, functional involvement of a majority of lncRNAs remains unclear, up to date. Even so, a variety of lncRNAs were well investigated and found to play critical regulatory roles during MSC differentiation, such as a recently identified lncRNA named PU.1‐as, a antisense transcript of the PU.1 gene, can prevent PU.1 mRNA translation by forming a mRNA/AS lncRNA duplex, consequently promotes adipogenesis of MSCs.^26^ Another well studied lncRNA is ANCR, which could negatively regulate the osteogenic differentiation, enforced overexpression of ANCR can inhibit osteogenesis through EZH2‐mediated transcriptional repression of RUNX2.^27^

In this study, we first identified HOXA‐AS2 as a positive regulator for osteogenesis of MSCs. Attenuation of HOXA‐AS2 significantly elevated the activity of NF‐κB signalling which was demonstrated to have an inhibitory effect on osteogenic differentiation of MSCs. Inhibition of NF‐κB dramatically enhanced the calcium deposition and alkaline phosphatase (ALP) activity. More importantly, we determined the underlying mechanism by which HOXA‐AS2 regulates osteogenic differentiation of MSCs and discovered HOXA‐AS2 can epigenetically mediate the expression of the osteogenic master transcription factor SP7 in a NF‐κB and HDAC2‐coordinated manner.

## 3 MATERIAL AND METHODS

### 3.1 Isolation and culture of MenSCs

The menstrual blood samples were collected from healthy female donors by menstrual cup during the first few days of menses. The samples were then mixed with equal volume PBS and subjected to standard Ficoll procedures within 72 hours. After centrifugation, the karyocytes and the deciduous endometrium suspending in buffy coat were transferred into a new tube and washed twice with PBS. Finally, the cell pellets were resuspended with complete DMEM medium supplemented with 10% foetal bovine serum (FBS), 100 U/mL penicillin and 100 mg/mL streptomycin and the medium was changed twice a week. All the menstrual blood derived used in this study was harvested with the informed consent of the donors and this study was approved by Ethics Committee of the Xinxiang Medical University.

### 3.2 Isolation and culture of UCMSCs

The umbilical cord vessels were removed in 0.9% normal saline following disinfection in 75% ethanol for 1 minute. The cord was cut into cubes of about 1 cm^3^. After removal of the supernatant fraction, the precipitate was rinsed with DMEM and then centrifuged at 250 × *g* for 5 minutes. The tissue was digested with collagenase II at 37°C for 1 hour and further treated with 0.25% trypsin at 37°C for 0.5 hour. To neutralize the excess trypsin, FBS was then added to the mesenchymal tissue. The dissociated umbilical cord mesenchymal stem cells (UCMSCs) were resuspended with DMEM medium and counted using a haemocytometer. The live cells were then plated in a 6‐well culture plate at a density of 1 × 10^6^ cells per well and the medium was changed twice a week. All the UCMSCs used in this study were harvested with the informed consent of the donors and this study was approved by Ethics Committee of the Xinxiang Medical University.

### 3.3 Osteogenic induction of MSCs

To induce osteogenic differentiation of MenSCs and UCMSCs, the culture medium was removed when the cells grow to 60% confluency and then replaced by the osteogenic medium (Sigma‐Aldrich, Beijing, China) containing α‐MEM with 10% FBS, 10 mmol/L β‐glycerol phosphate, 10 nmol/L dexamethasone and 50 μg/mL ascorbic acid phosphate. The medium was changed every 2 days.

### 3.4 Alizarin Red S staining and ALP activity detection

For Alizarin Red staining, cells were fixed in 70% ethanol for 30 minutes and then subjected to 1% Alizarin‐Red solution for 1 minute. Images of the stained cells were scanned and captured. For ALP staining, cells were fixed with 70% ethanol for 30 minutes and then incubated with the BCIP/NBT liquid substrate system at 37°C for 30 minutes. Images of the stained cells were scanned and captured.

### 3.5 Antibodies and reagents

Anti‐p65 antibody (#sc‐109) was purchased from Santa Cruz (Santa Cruz, CA, USA), anti‐GAPDH (#2118), anti‐ac‐310 RelA (#3045s) antibodies were from Cell Signaling Technology (Beverly, MA, USA) and anti‐HDAC2 (#12922‐3‐AP) antibodies were purchased from Proteintech Group Inc (Wuhan, China). The chemical reagent Bay 11‐7082 (#B5556), Alizarin Red S (#A5533) and BCIP/NBT liquid substrate system (#B1911) were all obtained from Sigma (St Louis, MO, USA).

### 3.6 Plasmid constructions and lentiviral infection

The short hairpin RNAs (shRNAs) targeting human HOXA‐AS2 and RelA RNAs were cloned into a modified pLV‐H1‐Puro lentiviral vector. The corresponding sequences for these shRNAs were: shHOXA‐AS2, 5′‐AAACCTTGTAGATAGCTTGAGCTGG‐3′ shRelA, 5′‐CAA GATCAATGGCTACACA‐3′. The human HOXA‐AS2, amplified using reverse transcription PCR, was inserted into a modified pLV‐EF1α lentiviral vector as previously described.^28^ For lentiviral infection, experimental procedures were conducted as previously described.^28^

### 3.7 Quantitative RT‐PCR

Total RNAs were isolated from cells using Trizol reagent according to manufacturers’ instructions. Reverse transcription was performed with 1 μg total RNA using a qPCR RT Kit (# FSQ‐201) purchased from TOYOBO (Shanghai, China). Real‐time quantitative PCR was performed with an EvaGreen qPCR Master Mix from Applied Biological Materials Inc (Richmond, Canada). The relative changes of gene expression were determined by the 2^−ΔΔCT^ method. The primer sequence used in qRT‐PCR analysis for RUNX2 was F: 5′‐GGACGAGGCAAGAGTTTCAC‐3′, R: 5′‐GAGGCGGTC AGAGAACAAAC‐3′; for SP7 was F: 5′‐CACAGCTCTTCTGACTGTCTG‐3′, R: 5′‐CTGGTGAAATGCCTGCATGGAT‐3′; for SPP1 was F: 5′‐AGCCAATGATGAG AGCAATG‐3′, R: 5′‐TCCTTACTTTTGGGGTCTAC‐3′; for GAPDH was F: 5′‐CATGAGAAGTATGACAACAGCCT‐3′, R: 5′‐AGTCCTTCCACGATACCAAAGT‐3′.

### 3.8 Chromatin immunoprecipitation

Briefly, 10^7^ HUVEC cells were cross‐linked with 1% formaldehyde for 10 minutes at room temperature and then quenched with glycine at the concentration of 125 mmol/L. After washing two times with PBS, the cells were lysed by a lysis buffer (50 mmol/L Tris‐HCl pH7.5, 150 mmol/L NaCl, 1 mmol/L EDTA, 1% Triton X‐100, 0.1%SDS, 0.5 mmol/L PMSF), subsequently sonicated with a sonicator so that the chromatin DNA was sheared into fragments from 100 to 500 bp. The sonicated lysates were then cleared and incubated with indicated antibodies for 6 hours at 4°C. Thirty microlitre protein A beads were added and incubated for 1 hour at 4°C with gentle rotation. Immunoprecipitates were washed three times with the lysis buffer and one time with a high salt buffer (50 mmol/L Tris‐HCl pH 7.5, 300 mmol/L NaCl, 1 mmol/L EDTA, 1% Triton X‐100, 0.1% SDS, 0.5 mmol/L PMSF). DNA was eluted in elution buffer (100 mmol/L NaHCO3, 1% SDS) and cross‐links were reversed overnight. RNA and protein were digested using RNase A and Proteinase K respectively. The immunoprecipitated DNAs were determined by qRT‐PCR. The SP7 primer sequences used in chromatin immunoprecipitation (ChIP) assays were F: 5′‐GTTGATGGGAAGCTCAGGTC‐3′, R: 5′‐AGA TAGGCAAGGGGACCCTC‐3′.

### 3.9 Statistical analysis

Three different MSC batches were used to do individual experiments in this study. Student's *t* test was used for comparisons between two groups. *P* < 0.05 was considered statistically significant. All data are representative of at least three independent experiments and presented as mean ± SD.

## 4 RESULTS

### 4.1 HOXA‐AS2 expression is significantly increased during osteogenic induction

**Figure 1:**
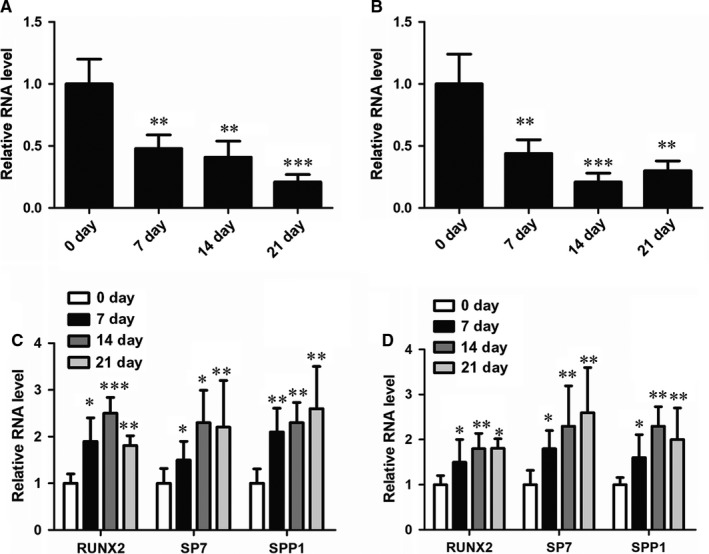
The expression of HOXA‐AS2 is downregulated upon osteogenic induction. A, qRT‐PCR analysis in MenSCs showing the RNA expression level of HOXA‐AS2 under the condition of osteogenic media induction for 0, 7, 14 and 21 d. B, qRT‐PCR analysis in UCMSCs showing the RNA expression level of HOXA‐AS2 under the condition of osteogenic media induction for 0, 7, 14 and 21 d. C, qRT‐PCR analysis in MenSCs showing the mRNA expressions of osteogenic marker genes under the condition of osteogenic media induction for 0, 7, 14 and 21 d. D, qRT‐PCR analysis in UCMSCs showing the mRNA expressions of osteogenic marker genes under the condition of osteogenic media induction for 0, 7, 14 and 21 d. All values are the average of at least three biological replicates and data shown are the mean ± SD. *P < 0.05, **P < 0.01, ***P < 0.001 vs 0 d

It is well known that inflammation is critically associated with osteogenesis. Moreover, recent studies showed the lncRNA HOXA‐AS2 plays a regulatory role in inflammation‐linked cancers.^29^, ^30^, ^31^ Therefore, we suggested HOXA‐AS2 is involved in the regulation of osteogenic differentiation. To test this hypothesis, we monitored the dynamic change of HOXA‐AS2 expression in menstrual blood‐derived mesenchymal stem cells (MenSCs) and UCMSCs during osteogenic induction by qRT‐PCR analysis. As a consequence, we found the expression of HOXA‐AS2 is dramatically reduced in both MenSCs and UCMSCs upon osteogenic media induction (Figure 1A,B), indicating HOXA‐AS2 may act as a crucial regulator participating in the osteogenic regulation. To verify the osteogenic differentiation after specific induction, we also measured the RNA expression levels of osteogenesis‐associated marker genes and we found that RUNX2, SP7 and SPP1 all displayed increased expressions after osteogenic induction (Figure 1C,D), which is consistent with the prior findings.

### 4.2 Inhibition of HOXA‐AS2 induces osteogenesis

**Figure 2:**
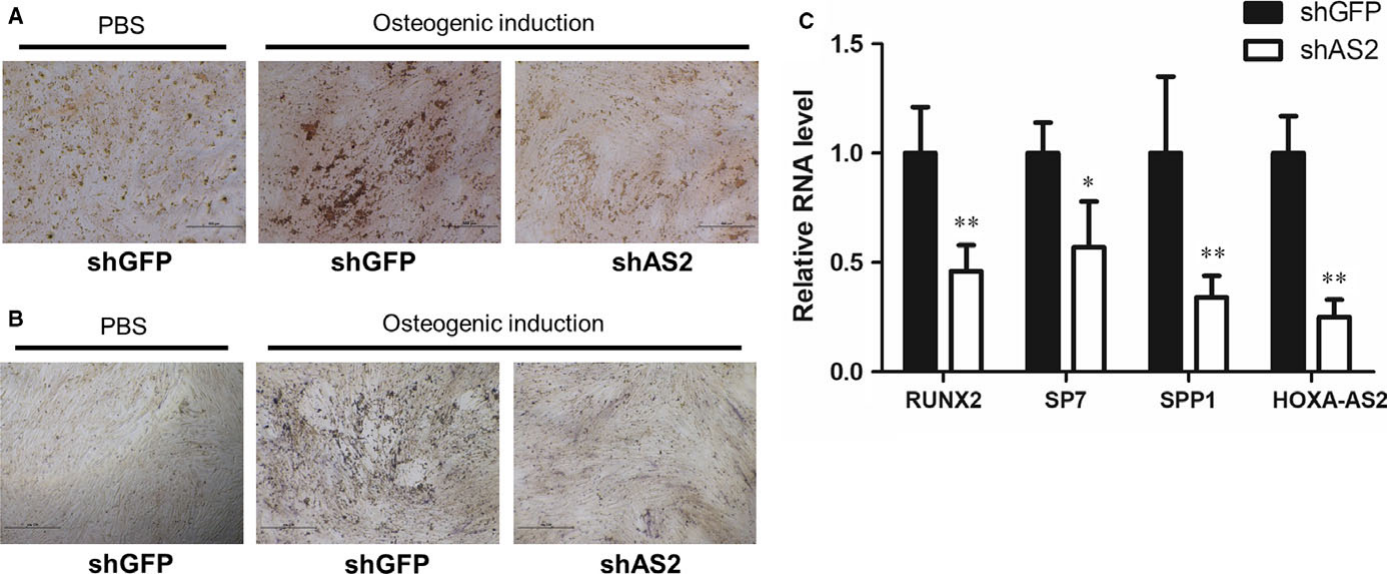
The expression of HOXA‐AS2 is downregulated upon osteogenic induction. A, The effect of HOXA‐AS2 on calcium deposition in MenSCs, determined by Alizarin Red S staining. B, The alkaline phosphatase activity was examined in MenSCs by BCIP/NBT liquid substrate system, with or without HOXA‐AS2 knockdown. C, The effect of HOXA‐AS2 on osteogenic marker gene expressions in MenSCs, measured by qRT‐PCR. All values are the average of at least three biological replicates and data shown are the mean ± SD. *P < 0.05, **P < 0.01 vs shGFP

To determine the regulatory role of HOXA‐AS2 in osteogenic differentiation, we performed Alizarin Red S staining in both MenSCs and UCMSCs, with or without HOXA‐AS2 knockdown, to observe the effect of HOXA‐AS2 on calcium deposition. The Alizarin Red S staining analysis showed that attenuation of HOXA‐AS2 can markedly repress the osteogenic media‐induced calcium deposition in both of the two cell lines (Figure 2A and Figure S1A). To further confirm this data, we measured the activity of ALP in control and HOXA‐AS2‐depleted MenSCs, respectively, and found HOXA‐AS2 depletion has an inhibitory effect on ALP activity, compared to the control sample (Figure 2B). Furthermore, we examined the expressions of osteogenesis‐associated marker genes when HOXA‐AS2 was silenced. Consequently, the expressions of the osteogenic marker genes RUNX2, SP7 and SPP1 were significantly downregulated in both MenSCs and UCMSCs with HOXA‐AS2 knockdown (Figure 2C and Figure S1B).

### 4.3 HOXA‐AS2 regulates the transcriptional activity of NF‐κB

**Figure 3:**
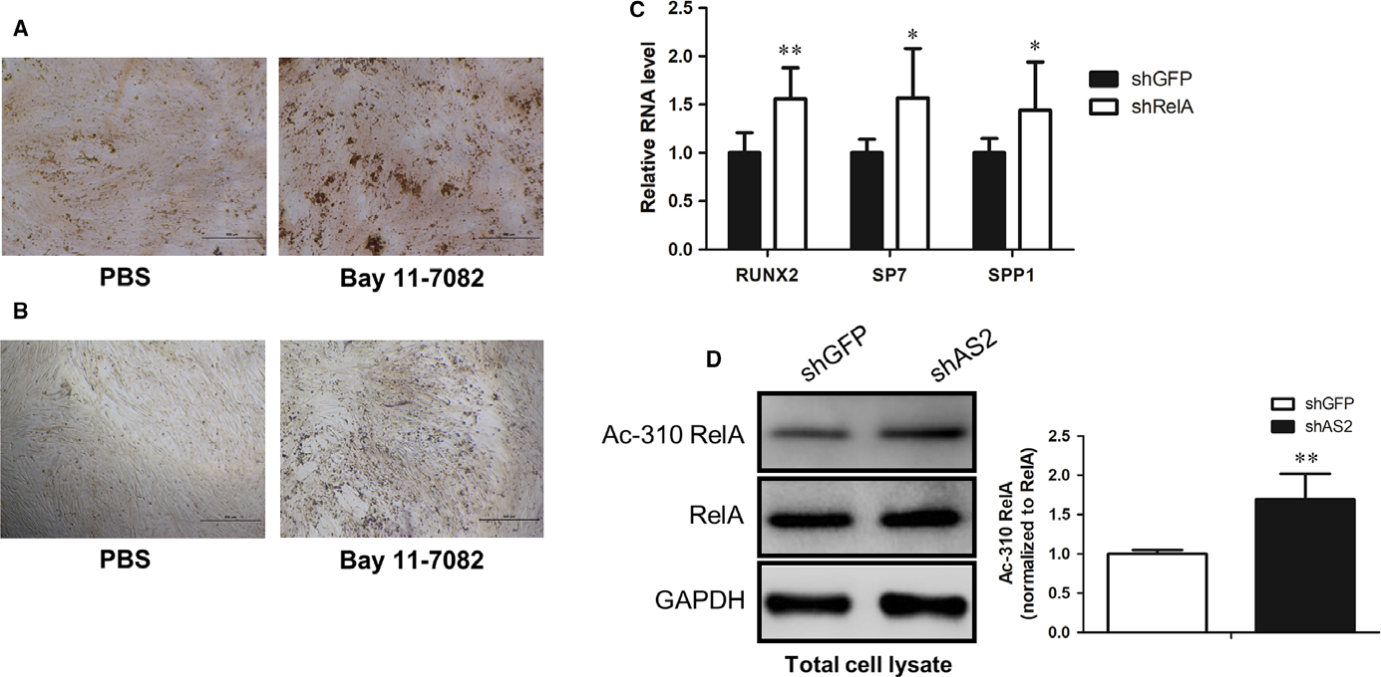
HOXA‐AS2 regulates the transcriptional activity of NF‐κB. A, The effect of Bay 11‐7082 on calcium deposition in MenSCs, determined by Alizarin Red S staining. B, The effect of Bay 11‐7082 on alkaline phosphatase activity in MenSCs, examined by BCIP/NBT liquid substrate system. C, The expressions of osteogenic marker genes were examined by qRT‐PCR in control and HOXA‐AS2‐depleted MenSCs respectively. D, Western blot analysis showing the effect of HOXA‐AS2 on acetylation status of RelA K310 site in MenSCs with or without HOXA‐AS2 knockdown, and then quantified using ImageJ software. All values are the average of at least three biological replicates and data shown are the mean ± SD. *P < 0.05, **P < 0.01 vs shGFP

Prior studies reported the primary inflammatory signalling NF‐κB is involved in the regulation of osteogenesis.^18^, ^32^ Moreover, HOXA‐AS2 was shown to play a regulatory role in inflammation‐linked cancers. Based on these investigations, we suggested that HOXA‐AS2 regulates the osteogenic differentiation, at least in part, through controlling the activity of NF‐κB signalling. To prove this hypothesis, we first assayed the role of NF‐κB in the regulation of mesenchymal stem cell (MSC) osteogenesis. We found the NF‐κB inhibitor, Bay 11‐7082 markedly induced the calcium deposition and ALP activity of MenSCs (Figure 3A,B). To confirm the inhibitory effect of NF‐κB on osteogenesis, we employed lentivirus‐mediated shRNA knockdown of RelA, a trans‐activating subunit of NF‐κB, and then determined the expressions of osteogenic marker genes after HOXA‐AS2 knockdown in MenSCs. The qRT‐PCR analysis revealed that attenuation of RelA significantly elevated the expressions of RUNX2, SP7 and SPP1 (Figure 3C), which is identical to the effect of Bay 11‐7082 on osteogenesis. Collectively, these results further validate the inhibitory role of NF‐κB signalling in osteogenic differentiation of MSCs.

To examine the interplay between HOXA‐AS2 and NF‐κB, we measured the K310 acetylation status of RelA, widely accepted as the key modification site for transcriptional activity of NF‐κB, after depleting HOXA‐AS2 in MenSCs. Western bolt analysis showed the K310 acetylation level was dramatically increased in HOXA‐AS2‐depleted MenSCs, compared with the shGFP‐treated control sample (Figure 3D). In order to further validate the regulatory role of HOXA‐AS2 in the regulation of NF‐κB activity, we performed HOXA‐AS2 overexpression in MenSCs, using a lentiviral vector and then detected the effect of overexpressed HOXA‐AS2 on RelA K310 acetylation status. As expected, aberrant overexpression of HOXA‐AS2 was found to obviously inhibited the K310 acetylation level of RelA (Figure S2). Taken together, these findings suggest that HOXA‐AS2 functions as a critical repressor for the activity of NF‐κB signalling.

### 4.4 HOXA‐AS2 epigenetically regulates SP7 expression by a NF‐κB and HDAC2‐coordinated mechanism

**Figure 4:**
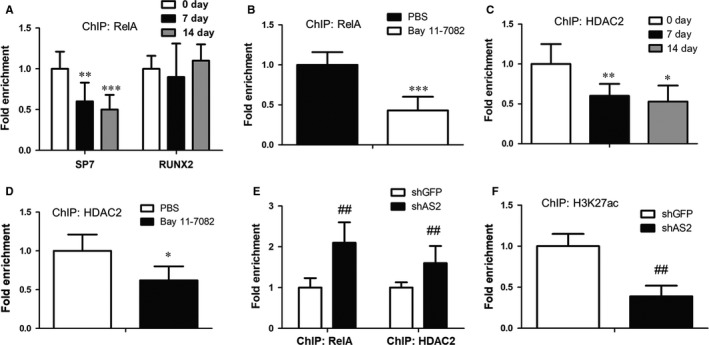
HOXA‐AS2 regulates expression of the master transcription factor SP7. A, Anti‐RelA ChIP assay showing the distribution of NF‐κB at SP7 promoter region during osteogenic induction of MenSCs. B, Anti‐RelA ChIP assay showing the effect of Bay 11‐7082 on the distribution of NF‐κB at SP7 promoter in MenSCs. C, The binding ability of HDAC2 at SP7 promoter was measured in MenSCs by ChIP assay, with an antibody against HDAC2. D, The effect of Bay 11‐7082 on chromatin binding of HDAC2 at SP7 promoter, determined by anti‐HDAC2 ChIP assay in MenSCs. E, Anti‐RelA and anti‐HDAC2 ChIP assays were performed in MenSCs, with or without HOXA‐AS2 konckdown. F, ChIP assay was performed in MenSCs, with an antibody against H3K27ac, to measure the effect of HOXA‐AS2 on K27 site acetylation of histone H3. All values are the average of at least three biological replicates and data shown are the mean ± SD. *P < 0.05, **P < 0.01, ***P < 0.001 vs 0 d or PBS; ^##^ *P* < 0.01 vs shGFP

Given the crucial role of HOXA‐AS2 in the regulation of osteogenic differentiation, we sought to determine the underlying mechanism by which HOXA‐AS2 regulate osteogenesis. As shown in Figure 3, HOXA‐AS2 can modulate NF‐κB activity. Moreover, NF‐κB can mediate the expressions of osteogenesis‐associated markers. Thus, we speculate whether HOXA‐AS2 could mediate expressions of the master transcription factor RUNX2 or SP7 in a NF‐κB‐dependent manner. To prove this hypothesis, we first performed ChIP assay, with an antibody against RelA, after osteogenic induction in MenSCs, to observe the binding ability of NF‐κB at RUNX2 and SP7 promoter regions respectively. The ChIP results revealed the distribution of NF‐κB at SP7 promoter was markedly abolished upon osteogenic induction (Figure 4A). However, the RelA occupancy at RUNX2 promoter region almost had no alteration when exposed to osteogenic induction media. Furthermore, the NF‐κB inhibitor Bay 11‐7082‐treated MenSCs showed reduced RelA distribution at SP7 promoter, compared with a PBS‐treated control sample (Figure 4B). These data indicate NF‐κB can specially bind to SP7 promoter region and contribute to its transcription regulation.

Increasing evidence revealed NF‐κB is involved in the deacetylase HDAC2‐mediated histone H3 lysine 27 acetylation (H3K27ac).^33^, ^34^ Therefore, we suspected HOXA‐AS2 may regulate the SP7 expression in a NF‐κB‐mediate HDAC2 acetylation manner. To test this notion, we first measured the binding capacity of HDAC2 at SP7 promoter, using anti‐HDAC2 ChIP assay. As a consequence, HDAC2 distribution at SP7 promoter region was found to be significantly impaired after osteogenic induction (Figure 4C). Next, we asked whether the recruitment of HDAC2 to SP7 promoter depends on NF‐κB. Anti‐HDAC2 ChIP was carried out in MenSCs with or without Bay 11‐7082 treatment. The result showed Bay 11‐7082‐treated cells obviously abrogated HDAC2 occupancy at SP7 promoter, relative to PBS‐treated control sample (Figure 4D). More importantly, we conducted HOXA‐AS2 knockdown in MenSCs and then examined the effect of HOXA‐AS2 on NF‐κB and HDAC2 binding affinity with SP7 promoter, using anti‐NF‐κB and anti‐HDAC2 ChIP assays respectively. Interestingly, the ChIP results revealed inhibition of HOXA‐AS2 dramatically augmented both the NF‐κB and HDAC2 occupancies at SP7 promoter (Figure 4E). Furthermore, we analysed the H3 lysine 27 acetylation status which can be regulated by HDAC2 in control and HOXA‐AS2‐depleted MenSCs by ChIP assay, with an antibody against H3K27ac. Consequently, attenuation of HOXA‐AS2 displayed reduced acetylation level of histone H3 at K27 site (Figure 4F). These observations suggest that HOXA‐AS2 can regulate SP7 transcription through NF‐κB and HDAC2‐dependent H3K27ac regulation mechanism.

## 5 DISCUSSION

Mesenchymal stem cells, a source of adult stem/progenitor cells, are widely investigated as a critical cell model. More importantly, mesenchymal stem cells can serve as an extremely useful medical therapeutic to regenerate or repair a variety of mature tissues, especially the bone tissue, due to its multipotential differentiation capacities. However, the potential molecular mechanism underlying osteogenic differentiation remains largely unknown, which blocks the clinical treatment of tissue repair and regeneration. In this study, we characterized the lncRNA HOXA‐AS2 as a critical regulator for osteogenesis of mesenchymal stem cells. Inhibition of HOXA‐AS2 significantly impaired the calcium deposition and alkaline phosphatase activity. Consistently, the expressions of osteogenesis‐associated markers were repressed when HOXA‐AS2 was depleted. These findings emphasize the positive role of HOXA‐AS2 in the development of osteogenesis and offer us a significant therapeutic target for clinical investigation of the tissue repair and regeneration.

Previous studies have shown inflammation has a significant correlation with osteogenic differentiation.^18^, ^19^, ^20^ Nevertheless, the precise molecular basis has not been presented up to date. In the current study, we first examined the regulatory role of the primary inflammatory signalling NF‐κB in osteogenesis, using gain or loss of NF‐κB function experiments. Notably, we discovered inhibition of NF‐κB not only can induce the calcium deposition and alkaline phosphatase activity of MenSCs but also enhanced the expressions of osteogenic marker genes, suggesting the important inhibitory effect of NF‐κB on osteogenesis.

**Figure 5:**
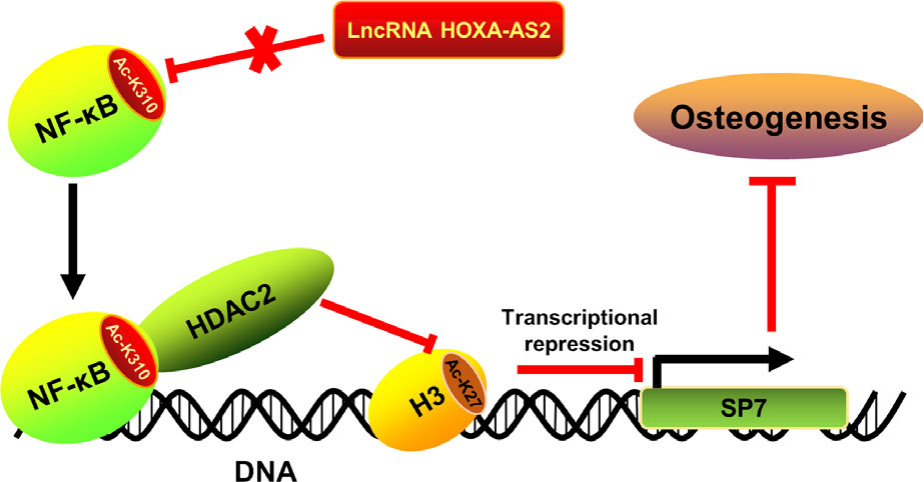
Schematic representation of a critical molecular mechanism by which HOXA‐AS2 regulates osteogenesis. HOXA‐AS2 plays an inhibitory effect on NF‐κB activity by repressing the K310 acetylation status of RelA. Once HOXA‐AS2 becomes dysfunctional, the NF‐κB will be activated and recruited to the promoter of master transcription factor SP7, co‐activating the deacetylase HDAC2. The HDAC2 will then contribute to the deacetylation of H3K27, consequently leading to the transcriptional repression of SP7

Based on the prior studies that HOXA‐AS2 is implicated in inflammation‐linked cancers,^29^, ^30^, ^31^ we suggested that HOXA‐AS2 regulates the osteogenic differentiation via mediating NF‐κB activity. Western blot analysis showed HOXA‐AS2 could suppress the transcriptional activity of NF‐κB by inhibiting K310 acetylation of RelA, which identified HOXA‐AS2 as a crucial regulator of NF‐κB activation. In the following investigations, we characterized the osteogenic master transcription factor SP7 as a transcriptional target of NF‐κB and found both NF‐κB and the deacetylase HDAC2 are enriched at the SP7 promoter region (Figure 5). Moreover, the ChIP assay suggested NF‐κB was able to help recruit HDAC2 to the SP7 promoter and facilitate the deacetylation of H3K27, eventually lead to the transcriptional repression of SP7 (Figure 5). Collectively, these findings suggest that the lncRNA HOXA‐AS2 could positively regulate the osteogenesis of MSCs by epigenetically mediating the master transcription factor SP7 expression. The functional importance of HOXA‐AS2 in osteogenic differentiation reveals it may become a promising therapeutic target for clinical bone tissue repair and regeneration.

## CONFLICT OF INTEREST

The authors declare that there is no conflict of interest.

## AUTHOR CONTRIBUTIONS

J.L. and X.Z. conceived and designed the project. X.Z. and J.Y. performed most of the experiments. X. Z wrote the manuscript. All authors contributed to discussions.
