# Supplementary material for: LncRNA HOXA‐AS2 positively regulates osteogenesis of mesenchymal stem cells through inactivating NF‐κB signalling
Source: J Cell Mol Med. 2018 Dec 8;23(2):1325–32. doi: 10.1111/jcmm.14034 (PMC6349193; doi:10.1111/jcmm.14034)
Supplement: Supplementary file 1 [file JCMM-23-1325-s001.docx]

**Supplementary data**


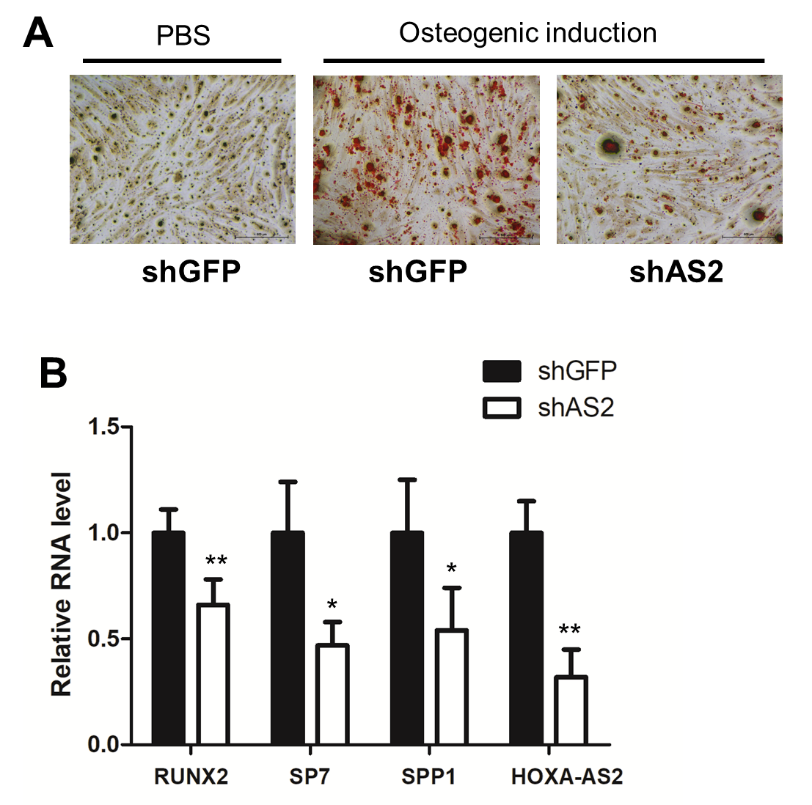


Supplementary Figure S1. **A**) The effect of HOXA-AS2 on calcium deposition in UCMSCs, determined by Alizarin Red S staining. **B**) The effect of HOXA-AS2 on osteogenic marker gene expressions in UCMSCs, measured by qRT-PCR.

All values are the average of at least 3 biological replicates and data shown are the mean±SD. *P<0.05, **P<0.01 versus shGFP.


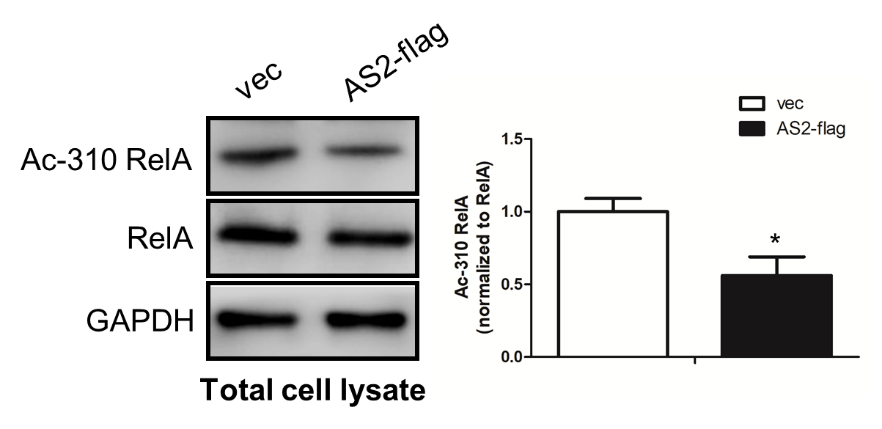


Supplementary Figure S2. The effect of overexpressed HOXA-AS2 on K310 acetylation status of RelA. MenSCs were infected with control and HOXA-AS2 overexpression vectors, respectively, subsequently subjected to western blot and quantified by using ImageJ software. All values are the average of at least 3 biological replicates and data shown are the mean±SD. *P<0.05 versus vec.
